# Calling, Courtship, and Condition in the Fall Field Cricket, *Gryllus pennsylvanicus*

**DOI:** 10.1371/journal.pone.0060356

**Published:** 2013-03-20

**Authors:** Sarah J. Harrison, Ian R. Thomson, Caitlin M. Grant, Susan M. Bertram

**Affiliations:** Department of Biology, Carleton University, Ottawa, Ontario, Canada; California State University Fullerton, United States of America

## Abstract

Theoretically, sexual signals should provide honest information about mating benefits and many sexually reproducing species use honest signals when signalling to potential mates. Male crickets produce two types of acoustic mating signals: a long-distance mate attraction call and a short-range courtship call. We tested whether wild-caught fall field cricket (*Gryllus pennsylvanicus*) males in high condition (high residual mass or large body size) produce higher effort calls (in support of the honest signalling hypothesis). We also tested an alternative hypothesis, whether low condition males produce higher effort calls (in support of the terminal investment hypothesis). Several components of long-distance mate attraction calls honestly reflected male body size, with larger males producing louder mate attraction calls at lower carrier frequencies. Long-distance mate attraction chirp rate dishonestly signalled body size, with small males producing faster chirp rates. Short-range courtship calls dishonestly reflected male residual mass, as chirp rate and pulse rate were best explained by a curvilinear function of residual mass. By producing long-distance mate attraction calls and courtship calls with similar or higher effort compared to high condition males, low condition males (low residual mass or small body size) may increase their effort in current reproductive success at the expense of their future reproductive success, suggesting that not all sexual signals are honest.

## Introduction

Honest signalling was termed “ubiquitous” in a recent game theory paper modeling signal evolution [1]. Biological signals are considered honest when they provide useful information to the receiver [2]. More specifically, sexual signals are considered honest when they indicate the potential benefits that a female could receive by mating with an advertising male [3]–[10]. Sexual signals may indicate mating benefits because (1) preferred signals are often costly to produce; (2) only males good at acquiring nutrients or using them efficiently may be able to support the costs of signal production; and (3) males with more nutrients may be of higher fitness or may be able to invest more in providing benefits to females. Males that are able to obtain more nutrients may have greater energy stores, and several studies have found a positive relationship between dietary nutrient availability and sexual signalling [11]–[16]. Given these points, females may benefit from selecting mates that exhibit condition-dependent signals. Here condition is defined as variation in resource acquisition ability [17], which may result from differences in resource availability in the environment and/or individual physiological differences in the ability to assimilate and utilize resources.

The cost of producing sexual signals is often dependent on available nutrients, which in turn is subject to life-history trade-offs. Allocating nutrients to sexual signalling must, therefore, be balanced against the nutrients required for growth and survival [8], [18], [19]. High condition males with an abundance of nutrients may be better able to afford the costs of allocating resources to sexual signalling than poor condition males (i.e. honest signalling [20]). Alternatively, poor condition males with reduced future reproductive potential may allocate more nutrients towards sexual signalling, thereby maximizing their current reproductive success at the expense of their future reproductive success (terminal investment hypothesis) [21]–[25]. When this occurs, one should see sexual signals being unreliable indicators of condition. There are several examples of poor condition males signalling to females with higher effort than high condition males [22], [26]–[29]. Game theory models reveal that dishonest sexual signals can be maintained provided the signals are honest on average, and the frequency of cheaters is low enough that receivers are more often likely to benefit from trusting that signals are honest [21], [25].

Here we investigate whether male sexual signals are honest indicators of condition, using body size and residual mass as proxies. Male field crickets (Gryllinae) rub their forewings together to produce two types of multicomponent acoustic signals (calls) to attract and court potential mates [30]. Males produce a long-distance mate attraction call to broadcast to distant females. Once they come into physical contact with a female they switch to quieter short-range courtship calls [30]. Variation in long-distance mate attraction calls influences male mating success, as females tend to phonolocate towards males that call most often (*Gryllus integer*: [31]; *G. campestris*: [32]; *Teleogryllus commodus*: [33], [34]), with higher chirp rates and longer chirp durations (*G. lineaticeps*: [35]), and longer signalling bout durations (*G. integer*: [36], [37]). While comparatively little is known about female preference for short-range courtship calls, female crickets appear to prefer courtship calls with higher chirp rates (*G. lineaticeps*: [38]), higher tick rates with longer durations of higher frequency ticks (*G. bimaculatus*: [39]), and higher sound rates with longer chirp, pulse, and trill durations (*T. oceanicus*: [40]). Assuming that females base their mating decisions on both long-distance mate attraction calls and short-range courtship calls, males may use these calls to honestly convey possible mating benefits to potential mates.

While several studies have shown long-distance mate attraction calls to be nutrition-dependent (e.g. [12]–[14], [27], [32], [41]–[43]), short-range courtship calls have generally been found to not be nutrition dependent (e.g. [23], [38]). This lack of support for courtship calling nutrition-dependence may result from (1) less rigorous examinations of the fine scale temporal aspects of courtship calls compared to long-distance mate attraction calling studies, (2) experimental diets not reflecting natural feeding regimes, (3) high breeding densities of laboratory-reared crickets altering selection pressures on male calls, or (4) males in poor condition artificially inflating their calls to maximize their current reproductive success at the expense of their future reproduction.

We use fall field crickets (*Gryllus pennsylvanicus*) to test two alternative hypotheses: (1) whether males in high condition (high residual mass or large body size) produce higher effort calls in support of the hypothesis that males signal honestly; and (2) whether low condition males (low residual mass or small body size) produce higher effort calls in support of the terminal investment hypothesis that low condition males maximize their current reproductive output [24]. We used wild-caught crickets in an attempt to circumvent potential downfalls associated with laboratory-reared crickets, such as artificial feeding regimes that test only the effect of resource abundance, not the ability to acquire resources in a natural environment. Wild-caught crickets that vary in body size and residual mass allow us to explore the effect of natural variation in resource abundance and resource acquisition ability experienced during development in the wild. We quantified the variance in sexual signalling within and between individuals, determined whether long-distance mate attraction and short-range courtship calls were correlated, and examined the condition-dependent nature of these signals. Mate attraction calls and courtship calls were highly repeatable but largely uncorrelated. Our findings reveal partial support for both hypotheses. In support of the honest signalling hypothesis large males produced louder long-distance mate attraction calls at lower carrier frequencies than small males. In support of the terminal investment hypothesis small males called to attract mates from a distance using faster chirp rates than large males. Additionally, lean (low residual mass) males produced courtship calls with pulse and chirp rates equivalent to plump (high residual mass) males. Our results suggest that not all sexual signals are honest; low condition males might maximize their current reproductive success with higher signalling effort, possibly at the expense of future reproduction.

## Methods

### Ethics Statement

Our study was conducted in accordance with the guidelines of the Canadian Council on Animal Care.

### Collection and Husbandry

Adult *Gryllus pennsylvanicus* were collected at the Koffler Scientific Reserve (University of Toronto) at Jokers Hill in the Oak Ridges Moraine in King Township, north of Toronto, Ontario, Canada from 8 to 14 August, 2010 (no collecting permits required). Upon capture, adult crickets were individually housed in 520 mL clear plastic containers with crumpled unbleached paper towel for shelter and *ad libitum* water and food (powdered Harlan Teklad Inc. Rodent diet no. 8604M). Adults were transferred to Carleton University where they were housed in a temperature-controlled greenhouse at 28±2°C on a 14∶10 h light:dark cycle for a three day acclimation period.

### Long-Distance Mate Attraction Call Recording

**Figure 1:**
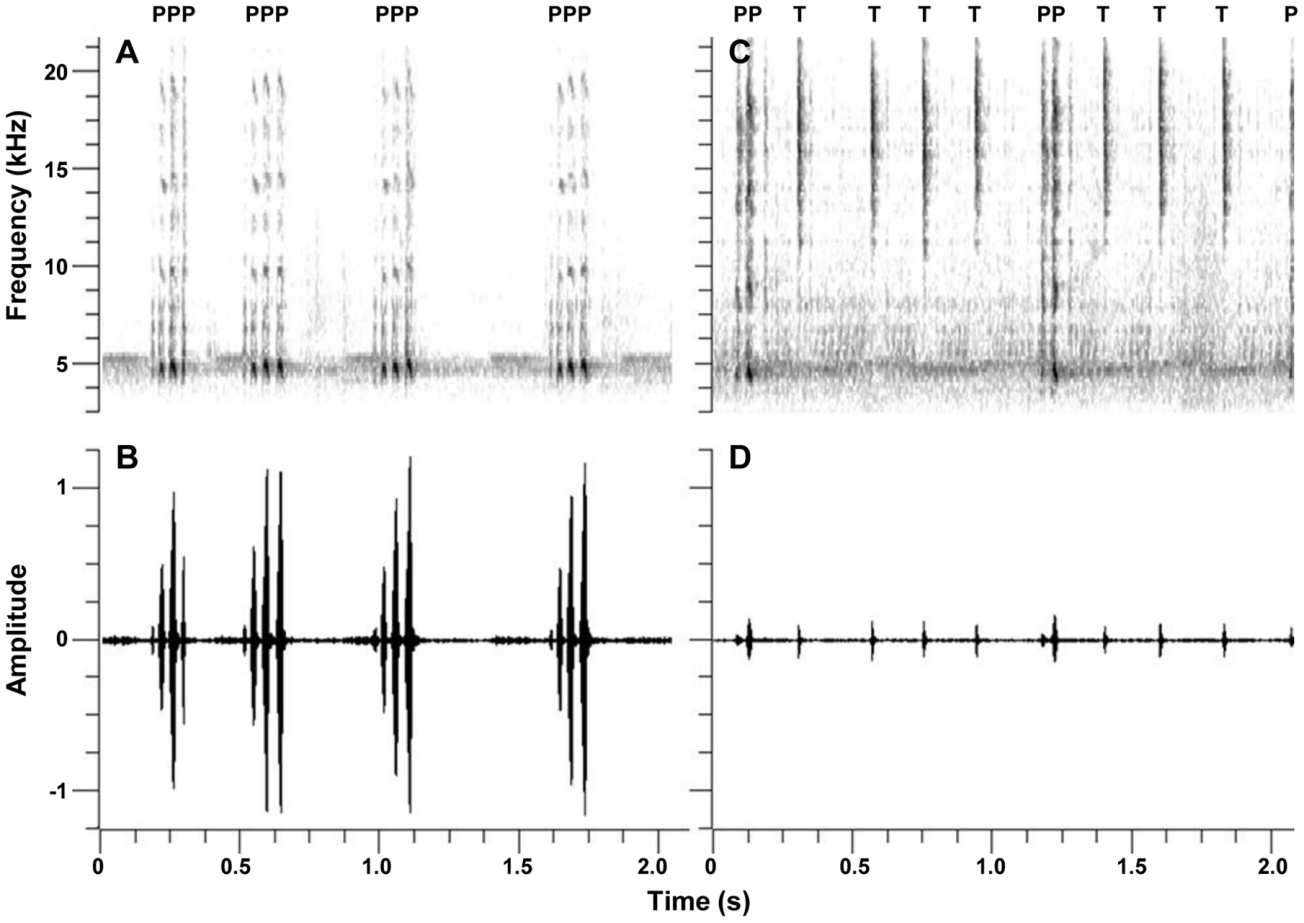
Long-distance mate attraction and courtship calls. Sonograms (top) and waveforms (bottom) of a *G. pennsylvanicus* long-distance mate attraction call (A & B) and a courtship call (C & D), showing the pulse (P) and tick (T) composition of each signal.

Male mate attraction calls were recorded for three days (72 h) immediately following acclimation to the Carleton University lab environment using the EARS II (Electronic Acoustic Recording System II; designed by Cambridge Electronic Design, Cambridge, UK). The EARS II is a system of 96 sound-proof Styrofoam boxes, each lined with acoustic foam to avoid sound contamination by neighbouring crickets, that simultaneously records and monitors all mate attraction calling of individual crickets (for further details refer to [27]). Each box contains a microphone and an LED light set to the same 14∶10 h light:dark cycle as the acclimatization room. The EARS II CricketSong software (Cambridge Electronic Design, Cambridge, UK) automatically filters out background noise and auto-adjusts its amplitude threshold for quiet or loud individuals. Male *G. pennsylvanicus* long-distance mate attraction calls are characterized by a series of ∼4.7 kHz pulses concatenated into chirps with ∼2–4 pulses per chirp (Figure 1 A & B; Table 1). Using the EARS II system we recorded nine fine scale temporal components of long-distance mate attraction calls [mean daily: pulse duration (ms), interpulse duration (ms), pulses per chirp, chirp duration (ms), interchirp duration (ms), call amplitude (dB), pulse carrier frequency (Hz), pulse rate (P/min), and chirp rate (Ch/min)] as well as three parameters indicative of calling effort [mean daily: number of pulses, number of chirps, and time spent calling]. Due to multicollinearity between several of these signal parameters, only six were used to characterize long-distance mate attraction calls in this study: chirp duration (ms), call amplitude (dB), pulse carrier frequency (Hz), pulse rate (P/min), chirp rate (Ch/min), time spent calling (min/day). Acoustic files were analyzed to produce a summary of mean calling parameters using Spike2 v6.12 (Cambridge Electronic Design, Cambridge, UK). Of the 62 males quantified, 60 produced mate attraction calls on all three days, while the remaining 2 produced calls on 2/3 days.

**Table 1:** Descriptive statistics for long-distance mate attraction call and courtship call parameters of 44 male *G. pennsylvanicus*, including coefficient of variation (CV) and repeatability (r) estimates.

Signal Type	Signal Parameter	Mean	SD	CV	r	F
**LD Mate Attraction**	Time Spent Calling (min/day)	254.67	164.30	64.51	0.65	6.34
	Chirp Rate (Ch/min)	68.50	18.88	27.57	0.79	12.35
	Pulse Rate (P/min)	1064.08	61.92	5.82	0.72	8.54
	Chirp Duration (ms)	110.96	13.07	11.78	0.77	11.02
	Pulse Carrier Frequency (Hz)	4684.20	183.47	3.92	0.76	10.07
	Amplitude (db)	61.67	8.94	14.50	0.73	9.12
**Courtship**	Chirp Rate (Ch/min)	111.75	31.58	28.26	0.66	4.84
	Tick Rate (T/min)	135.10	64.12	47.46	0.77	7.70
	Pulse Rate (P/min)	207.40	69.77	33.64	0.71	6.01
	Pulse/Tick Amp Ratio	1.85	1.96	106.12	0.87	14.04
	Pulse Carrier Frequency (Hz)	5018.12	247.57	4.85	0.94	31.70

**All repeatability estimates were significant at P<0.0001. LD Mate Attraction signals df  = 43,85; Courtship signals df  = 41,42.**

### Short-Range Courtship Call Recording

Males were placed with a random field-caught female immediately after being removed from the EARS II. Experimental adults were of unknown age and mating status, but are likely to have all been reproductively active because males were actively producing long-distance mate attraction calls just prior to collection, and females were collected in the vicinity of signalling males. Females received the same 72 h acclimation period as males, along with an additional 72 h period while male long-distance mate attraction calls were being recorded. Females had, therefore, been unmated for at least 6 days.

Courtship and mating trials were conducted between 1000 h and 2300 h over a two-day period. Each cricket pair was placed in a clear plastic 520 mL container without food or water. Courtship was observed continuously for 30 minutes. Using a handheld audio recorder (Handy Recorder H4, Zoom Corporation, Tokyo, Japan), we recorded at least 30 seconds of each male's short-range courtship calls immediately following initiation. A subset of males (N = 18) failed to produce courtship calls. This subset did not differ from other males in body size or residual mass and were excluded from all analyses.

Courtship calls were analyzed manually using Spike2 v. 6.12 (Cambridge Electronic Design, Cambridge, UK). *Gryllus pennsylvanicus* courtship calls are characterized by a series of chirps, each with 1–3 pulses at ∼5 kHz, intermixed with high frequency ticks with bandwidths ranging from ∼10–22 kHz (Figure 1 C & D; Table 1). Because female *G. pennsylvanicus* auditory receptors are most sensitively tuned to male acoustic calls at ∼5 kHz [44], and the pulse carrier frequency of male long-distance mate attraction calls is ∼4.7 kHz (Table 1), we ran courtship recordings through a high pass second order 4 kHz filter to reduce background noise.

Male's courtship calls were often interspersed with long-distance mate attraction calls. Courtship calls could unambiguously be distinguished from mate attraction calls due to their smaller number of pulses per chirp, the presence of high frequency ticks, and lower amplitude pulses. Because males often intermix call types during courtship we could not quantify the first courtship calls produced. Instead, we identified the first 15-seconds following a high frequency tick where the male continuously produced courtship calls without long-distance mate attraction calls. We subdivided this interval into three 5-second intervals and used the first and last 5-second intervals for analysis. By measuring two 5-second intervals we obtained two sets of short-range courtship calls, which we used to quantify repeatability (see below). We could not lengthen the interval between recordings because some females mounted males quickly.

Each male's two 5-second samples of courtship calling were analyzed for chirp rate (Ch/min), tick rate (T/min), pulse rate (P/min), pulse/tick amplitude ratio, and pulse carrier frequency (Hz). Although the mechanism is unknown, males occasionally produced a pulse and a tick simultaneously such that the amplitude of each separate element could not be measured. When this happened both the pulse and tick were included in the tick rate and pulse rate measurements but their amplitude was not included in the pulse/tick amplitude ratio measure. Two males produced only one short bout of short-range courtship calling before mating, so only a single sample (3 to 5 seconds) could be analyzed for these two individuals. These two males were excluded from the repeatability analyses.

### Condition Proxies

We define condition as variation in the ability to obtain, assimilate, and utilize nutritional resources. Therefore, we used body size and residual mass as proxies of condition. Male and female crickets were weighed to the nearest milligram following their mating trials using a Denver Instruments analytical balance (Pinnacle Series model PI-114; precision ±0.1 mg). Crickets were then photographed in a dorsal position using a Zeiss Discovery V12 stereo dissecting microscope (AxioVision v4.8, Carl Zeiss; magnification: ∼5x, resolution: ∼1.60 µm) from which pronotum area (mm^2^), width (mm), height (mm) and head capsule width (mm) was measured to the nearest micrometer. Male body size was quantified with a principal component analysis (PCA) to remove multicollinearity between the four size measurements. Size PC1 explained 91.8% of the variation (eigenvalue  = 3.67) and was loaded heavily by all measurements. Residual mass was calculated using a regression of body mass on body size (size PC1) [45], [46], a measure that appears to reflect energetic fat reserves to some degree in crickets [23].

### Statistical Analyses

All statistical analyses were performed in JMP v8.0.2 (SAS Institute, Cary, NC, USA). All acoustic call parameters except for mate attraction pulse rate and courtship pulse rate were Box Cox transformed to ensure they approximated normal distributions. To assess variability in acoustic calls among individual males we calculated the intraclass correlation coefficient, or repeatability (r), for long-distance mate attraction call parameters over three full days of recording. We quantified repeatability for short-range courtship call parameters to assess measurement error using our two five-second intervals. Repeatability was calculated as r  =  s^2^_A_/(s^2^+s^2^_A_) [47]. The among-groups (s^2^_A_) and within-group (s^2^) variance components were calculated from the mean squares from a one-way ANOVA as s^2^_A_ =  (Group MS – Error MS)/n_o_ and s^2^  =  Error MS, where n_o_, a coefficient related to the number of measurements for each male for each call component, was 2.00 for courtship call parameters and 2.94 for long-distance mate attraction call parameters [47].

We calculated the coefficient of variation (CV = 100* sd/|5|) for each mate attraction and courtship calling parameter across all males. We used pairwise correlations to quantify the relationships between long-distance mate attraction and short-range courtship traits. We used general linear models with a subset of the signalling traits to test whether signals honestly reflected condition. We used non-linear regression analyses to test whether males in poor condition (low residual mass or small size) maximized their current reproductive output. We corrected for multiple hypothesis tests using FDR_B-Y_ method [48].

## Results

### Acoustic Call Variability and Correlations

On average, male *G. pennsylvanicus* long-distance mate attraction calls were characterized by a series of 4.7 kHz pulses concatenated into 111 ms chirps with 2–4 pulses per chirp (Figure 1 A & B; Table 1). Pulse and chirp rates were 1064 pulses per minute and 69 chirps per minute, respectively, and males called for an average of 255 minutes per day at 62 db (Table 1). While all long-distance mate attraction call parameters were highly repeatable within each male's signals (Table 1; all r >0.65), time spent calling and chirp rate were highly variable across males, as evidenced by high coefficients of variation (Table 1; CV >27).

On average, male *G. pennsylvanicus* short-range courtship calls were characterized by a series of chirps at approximately 112 chirps per minute, each with 1–3 pulses at 5 kHz at a rate of 207 pulses per minute (Figure 1 C & D; Table 1). Courtship call pulses were generally louder than ticks, with an average pulse to tick amplitude ratio of 1.85 (Table 1). Pulses were intermixed with high frequency ticks having bandwidths ranging from approximately 10–22 kHz at an average rate of 135 ticks per minute (Figure 1 C & D; Table 1). All courtship call parameters were highly repeatable within each male's signals (Table 1; all r >0.66), usually with coefficients of variation indicating a high degree of variability across males (Table 1; CV >28), particularly for pulse to tick amplitude ratio (CV  = 106).

Short-range courtship call parameters were generally not significantly correlated with mate attraction calling parameters, with two exceptions: courtship pulse carrier frequency was positively correlated with long-distance mate attraction pulse rate (R = 0.493, P = 0.001) and pulse carrier frequency (Table 2; R = 0.765, P<0.001).

**Table 2:** Pairwise correlations between *G. pennsylvanicus* courtship call parameters (rows) and long-distance mate attraction call parameters (columns) showing p values for Pearson correlation coefficients.

		LD-Time Spent Calling (min/day)	LD-Chirp Rate (Ch/min)	LD-Pulse Rate (P/min)	LD-Chirp Duration (ms)	LD-Pulse Carrier Frequency (Hz)	LD-Amplitude (db)
**Court Chirp Rate** **(Ch/min)**	**R**	0.022	0.030	−0.192	0.264	−0.018	0.005
	**P**	0.886	0.847	0.211	0.084	0.907	0.972
**Court Tick Rate** **(T/min)**	**R**	−0.028	0.095	−0.033	0.071	−0.155	0.139
	**P**	0.859	0.539	0.832	0.647	0.316	0.368
**Court Pulse Rate** **(P/min)**	**R**	0.016	0.183	−0.135	0.334	−0.125	0.184
	**P**	0.917	0.236	0.381	0.026	0.418	0.232
**Court Pulse Carrier** **Frequency (Hz)**	**R**	0.289	−0.066	**0.493**	0.056	**0.765**	0.276
	**P**	0.057	0.668	**0.001**	0.717	**<0.001**	0.069
**Court Pulse/Tick** **Amplitude Ratio**	**R**	0.192	0.027	−0.057	0.295	−0.202	0.171
	**P**	0.211	0.864	0.711	0.052	0.188	0.267

**Significant correlations are indicated in bold (FDR_B–Y_ corrected alpha level of significance: P<0.013).**

### Condition and Acoustic Calling

Our general linear models examining whether call parameters honestly reflect condition suggest that long-distance mate attraction calls convey information about body size. Large males produced louder long-distance mate attraction calls, at lower carrier frequencies than small males (Table 3). Small males, however, produced long-distance mate attraction calls with faster chirp rates than large males (Table 3). Male long-distance call parameters did not convey significant information about residual mass. Similarly, male courtship call parameters did not convey significant information about body size or residual mass (Table 3).

**Table 3:** General linear models showing relationships between condition measures (body size and residual mass) and mate signalling traits (long-distance mate attraction and short-range courtship signals).

	Whole Model			Parameter Estimates			
Condition Measure	χ^2^	df	P	Model Parameters	Coefficient ± SE	χ^2^	P
**Body Size**	**19.818**	**6, 37**	**0.003**	LD-Time Spent Calling (min/day)	0.003±0.002	3.680	0.055
				LD-Chirp Duration (ms)	0.016±0.021	0.614	0.433
				**LD-Carrier Frequency (Hz)**	**−0.004±0.001**	**8.266**	**0.004**
				**LD-Amplitude (dB)**	**0.075±0.033**	**4.836**	**0.028**
				LD-Pulse Rate (P/min)	0.001±0.004	0.032	0.858
				**LD-Chirp Rate (Ch/min)**	**−0.046±0.015**	**8.915**	**0.003**
Body Size	7.417	5, 38	0.192	Court Chirp Rate (Ch/min)	0.004±0.013	0.099	0.754
				Court Tick Rate (T/min)	0.003±0.004	0.570	0.450
				Court Pulse Rate (P/min)	−0.001±0.006	0.050	0.823
				**Court Pulse/Tick Amplitude Ratio**	**0.354±0.140**	**5.991**	**0.014**
				Court Pulse Carrier Frequency (Hz)	−0.001±0.001	1.498	0.221
Residual Mass	4.903	6, 37	0.556	LD-Time Spent Calling (min/day)	0.028±0.028	1.016	0.314
				LD-Chirp Duration (ms)	−0.351±0.341	1.048	0.306
				LD-Carrier Frequency (Hz)	−0.033±0.023	2.073	0.150
				LD-Amplitude (dB)	0.346±0.547	0.397	0.529
				LD-Pulse Rate (P/min)	0.041±0.071	0.334	0.563
				LD-Chirp Rate (Ch/min)	−0.400±0.241	2.675	0.102
Residual Mass	10.364	5, 38	0.066	Court Chirp Rate (Ch/min)	−0.292±0.172	2.804	0.094
				**Court Tick Rate (T/min)**	**−0.115±0.056**	**3.966**	**0.046**
				Court Pulse Rate (P/min)	0.014±0.079	0.033	0.856
				Court Pulse/Tick Amplitude Ratio	2.725±1.877	2.059	0.151
				Court Pulse Carrier Frequency (Hz)	−0.009±0.015	0.387	0.534

**Significant overall models and individual model parameters are indicated in bold.**

**Figure 2:**
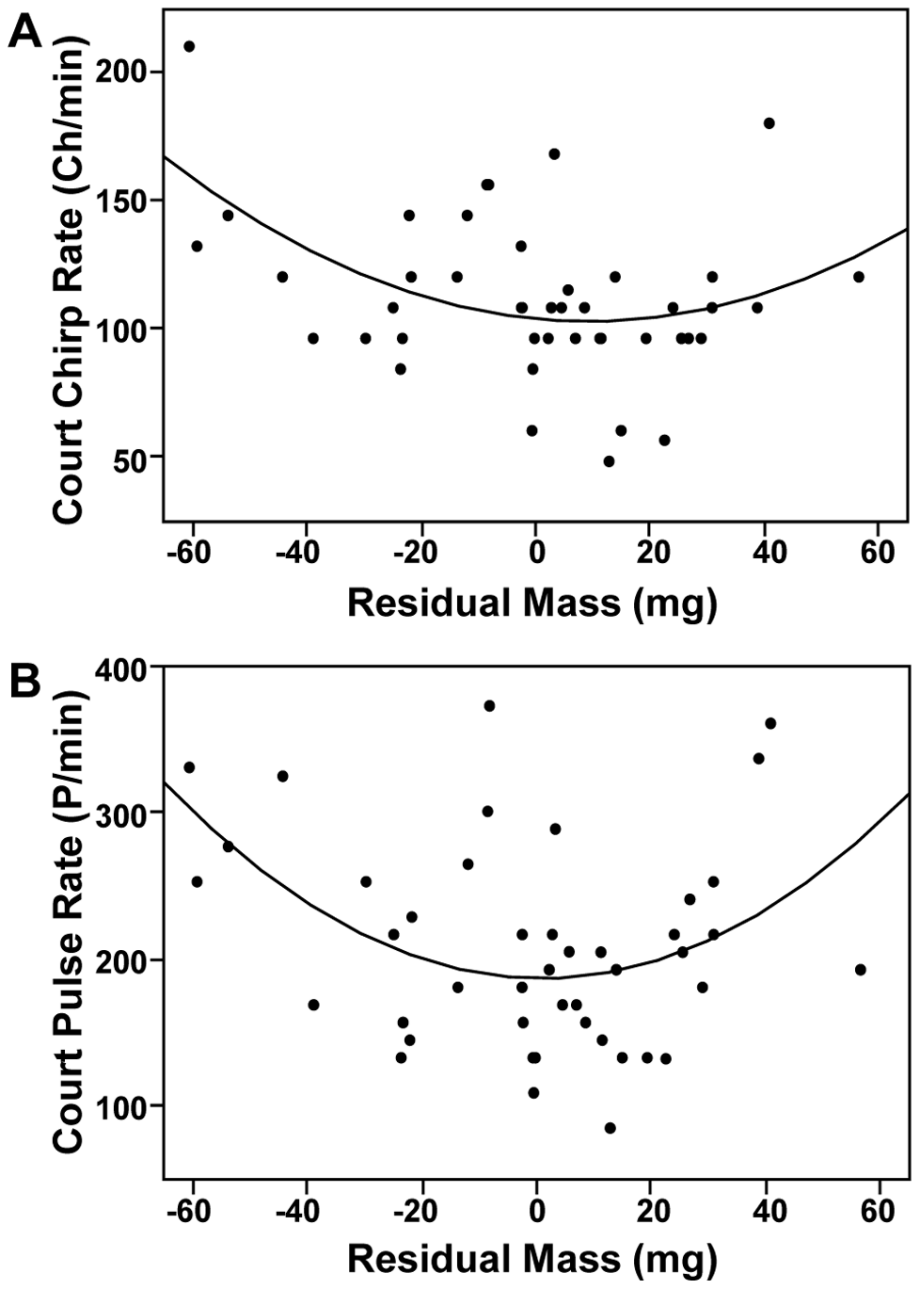
Non-linear relationship between residual mass and courtship call parameters. Residual mass predicts courtship chirp rate (top) and courtship pulse rate (bottom) where lean males (low residual mass) have chirp and pulse rates that are equivalent to plump males (high residual mass). Statistics are presented in Table 4.

Our non-linear regression models examining whether low condition males signal dishonestly revealed that lean males (low residual mass) courted females at rates equivalent to plump males (high residual mass). Lean males produced courtship calls with pulse rates and chirp rates equivalent to plump males, with males of intermediate residual mass having courtship calls with the lowest pulse and chirp rates (Table 4; Figure 2). All other non-linear regression models were not statistically significant and so were not included in Table 4.

**Table 4:** Models showing relationships between call parameters and linear/non-linear condition measures (only call parameters with significant models are shown).

	Whole Model			Parameter Estimates			
Call Parameter	χ^2^	df	P	Model Parameters	Coefficient ± SE	χ^2^	P
**Court Chirp Rate (Ch/min)**	**10.026**	**2, 41**	**0.007**	Residual Mass	−0.222±0.167	1.737	0.188
				Residual Mass * Residual Mass	**0.012±0.005**	**6.020**	**0.014**
**Court Pulse Rate (P/min)**	**9.345**	**2, 41**	**0.009**	Residual Mass	−0.073±0.372	0.038	0.845
				Residual Mass * Residual Mass	**0.031±0.010**	**8.259**	**0.004**

**Significant overall models and individual model parameters are indicated in bold.**

## Discussion

Theory predicts that male sexual signals should honestly indicate female mating benefits because the cost associated with signalling dishonestly should be too high for males in poor condition to maintain (the handicap principle; [5], [20], [49], [50]). Females may, therefore, benefit from selecting mates on the basis of condition-dependent signals. Cricket studies have largely supported this honest signalling theory, revealing that long-distance mate attraction calls are usually nutrition dependent (e.g. [12]–[14], [27], [32], [42], [43]).

In support of the honest signalling theory, we found that some signal components of field-captured male *G. pennsylvanicus*' long-distance mate attraction calls were indicative of male body size (Table 3). Similar to previous findings in other gryllid species [51], [52], larger *G. pennsylvanicus* males produced louder mate attraction calls at lower carrier frequencies (Table 3). Because large adult body size in field crickets is beneficial in aggressive contests between rival males over mating territories [53], [54], and several studies have shown body size to be heritable in crickets [55]–[58], females may secure good genes for offspring body size by selecting mates on the basis of their signalling amplitude and carrier frequency.

In contrast to honest signalling theory, the terminal investment hypothesis suggests that poor condition males with reduced future reproductive potential may increase their effort in sexual signalling in an attempt to secure a successful mating while they are still able [21]–[25]. While this hypothesis has received less attention in the sexual selection literature compared to the honest signalling hypothesis, several recent cricket studies have findings consistent with it. Smaller *Acheta domesticus* males transferred greater numbers of viable sperm to females than larger males [59]. *Gryllus assimilis* increased their signalling effort with increasing age, with older males producing higher pulse and chirp rates, and longer and louder chirps [60]. *Gryllus assimilis* males that experienced decreased body condition after being fed low quality diets called for more long-distance mate attraction bouts per night compared to males fed high quality diets [27]. Furthermore, males experiencing an immunological threat increased their investment in current reproduction through increased fighting success (*G. integer*: [29]), and faster chirp rates in mate attraction calls (*Allonemobius socius:* [28]).

In support of the terminal investment hypothesis, we found that small males produced long-distance mate attraction calls with higher chirp rates than large males (Table 3). Given that faster chirp rates are more energetically expensive to produce [61], and small body size may reflect poor nutritional resources and/or nutrient assimilation and utilization ability during juvenile development, chirp rate appears to be a dishonest signal of male condition. Small males may be overcompensating for their small size by chirping at faster rates in an attempt to attract females. Females may be able to detect this dishonest signal using information from other mating cues.

The question that remains, however, is what information are females gleaning from courtship calls? Courtship calls occur after females have located males and our findings suggest they also convey dishonest information about male condition. A curvilinear relationship exists between residual mass and courtship pulse and chirp rates such that both lean and plump males (low and high residual mass, respectively) courted females with similarly high pulse and chirp rates, with males of intermediate residual mass having the lowest pulse and chirp rates (Table 4; Figure 2). Lean males may be enhancing their courtship rates in an attempt to secure a successful mating. Enhanced investment in courtship may maximize male current reproductive success at the expense of future reproductive success [21]–[24].

Overall, our findings that (1) small males produced long-distance calls with faster chirp rates, and (2) males with low residual mass courted females with higher pulse and chirp rates than males of intermediate residual mass suggests the possibility of an alternative reproductive strategy in *G. pennsylvanicus.* Poor condition males may be increasing their investment in current reproduction at the expense of having fewer resources to devote to future reproduction or survival. Future studies should address the long-term consequences of courtship calling and longevity for males that vary in residual mass.

Given the high production costs of cricket acoustic calls, the use of multiple signals to attract a mate may seem maladaptive. However, multiple sexual signals may be adaptive by reducing female mate choice errors, providing different types of information on male quality and condition, or reducing time and energy spent assessing males [62]. We found long-distance mate attraction and short-range courtship calling parameters to be repeatable over time (Table 1), suggesting both call types have the potential to provide reliable information to females. Further, the relative lack of significant correlations between mate attraction and courtship calls (Table 2) suggests these two call types may convey distinct information to females (i.e. the multiple messages hypothesis [63]). Future studies examining sexual signalling in crickets should investigate relationships between multiple sexual cues in different sensory modalities in order to gain a better understanding of information being conveyed to females in these signals.

We interpret our findings with caution for several reasons. First, we have no information about male age or mating history, and these factors may influence behavioural tradeoffs in investing in current versus future reproductive effort. Similarly, males with low residual mass might have had less (or more) mating experience, resulting in enhanced courtship rates. Second, we assumed that males' residual mass reflected individual differences in resource acquisition. However, if low residual mass males are good at acquiring resources, they may risk investing much of their energy in signalling because they can easily replace it. Even if our assumption is valid that residual mass reflects individual differences in resource acquisition, our *ad libitum* feeding regime may have provided low residual mass males the resources necessary to enhance their courtship displays. In retrospect, a superior protocol would have been to weigh each cricket immediately following collection, then re-weigh them following *ad libitum* feeding to ascertain how residual mass changed. Given we only weighed crickets following *ad libitum* feeding, we have to assume that our protocol did not greatly alter the variation in condition determined by physiological differences in assimilation and utilization of resources between males.

Formal tests of the hypotheses that small males produce higher effort long-distance mate attraction calls and lean males court females with higher effort than plump males requires experimental manipulation of male condition using nutritionally explicit dietary treatments (i.e. geometric framework, [64]). That said, lab-based nutrient manipulation studies only test the effect of resource abundance on male signals, not the effect of resource acquisition ability in a natural environment. The benefit of using field-captured animals is that they naturally vary in residual mass, and are therefore likely to reflect variation in both resources acquisition ability and resource abundance.
